# Telodendrimers: Promising Architectural Polymers for Drug Delivery

**DOI:** 10.3390/molecules25173995

**Published:** 2020-09-02

**Authors:** Søren Mejlsøe, Ashok Kakkar

**Affiliations:** Department of Chemistry, McGill University, 801 Sherbrooke St. West, Montreal, QC H3A 0B8, Canada; smejlsoe@hotmail.com

**Keywords:** telodendrimer, dendrimers, linear polymers, hybrid structures, drug delivery, micelles, nanoformulations, soft nanoparticles, macromolecules

## Abstract

Architectural complexity has played a key role in enhancing the efficacy of nanocarriers for a variety of applications, including those in the biomedical field. With the continued evolution in designing macromolecules-based nanoparticles for drug delivery, the combination approach of using important features of linear polymers with dendrimers has offered an advantageous and viable platform. Such nanostructures, which are commonly referred to as telodendrimers, are hybrids of linear polymers covalently linked with different dendrimer generations and backbones. There is considerable variety in selection from widely studied linear polymers and dendrimers, which can help tune the overall composition of the resulting hybrid structures. This review highlights the advances in articulating syntheses of these macromolecules, and the contributions these are making in facilitating therapeutic administration. Limited progress has been made in the design and synthesis of these hybrid macromolecules, and it is through an understanding of their physicochemical properties and aqueous self-assembly that one can expect to fully exploit their potential in drug delivery.

## 1. Introduction

Macromolecules continue to offer exciting opportunities to develop functional nano-architectures to address key issues in areas, including biology [1,2,3]. There has been tremendous growth in their synthetic articulation from linear block-copolymers to branched and hyperbranched structures [4]. Amphiphilic diblock co-polymers, being the ones studied more extensively, have played a key role in assembling nanoformulations [5], in which lipophilic drugs could be physically encaged. Such soft nanoparticles have generally been very successful in providing solutions to challenges related to the solubility of active pharmaceutical agents in an aqueous medium (bioavailability), blood circulation times, and in reducing toxicities [6]. Branched miktoarm stars, with well-defined hydrophilic/hydrophobic components, provide a step-forward in enhancing the efficacy of their self-assembled micellar structures by lowering critical micelle concentrations, increasing drug pay-loads, and in controlling their release profiles [7,8]. The discovery of monodisperse and symmetric hyperbranched macromolecules (often referred to as dendrimers due to their tree-like growth) has further advanced the scope of macromolecules in biomedical applications [9]. This is attributed to their large surface area with plentiful end-groups for introducing a variety of functional groups, as well as their ability to act as unimolecular micelles. Each of these polymeric architectures has offered particular advantages in their own domain, and continue to constitute a topical area of research in drug delivery. Another more recent effort in enhancing the efficacy of therapeutic interventions is the design of hybrid structures, which could combine the advantageous features of linear/branched polymers with the hyperbranched dendrons. In addition to tuning the overall composition of the resulting self-assemblies, these architectural polymers, also called telodendrimers, could also provide a platform for combination therapy, in which a lipophilic drug could be covalently linked to the surface groups of the dendron, and another encapsulated in the resulting aqueous assemblies [10]. Telodendrimers, being a relatively new member of the macromolecular family, are academically exciting due to the role of their overall composition in tailoring the morphologies of the self-assemblies that result, and in finding better solutions to deliver drugs at disease sites.

**Figure 1:**
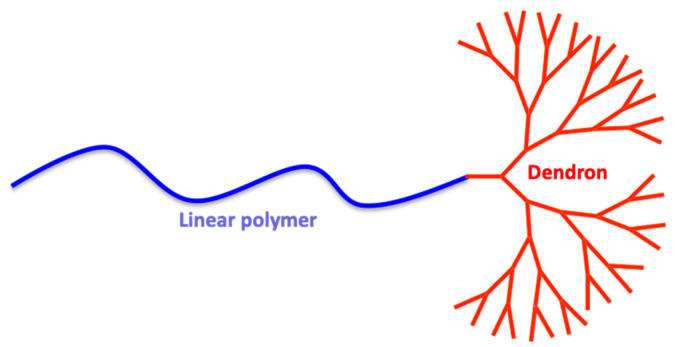
Basic structure of an architectural polymer (telodendrimer): Linear polymer and dendron hybrid.

The basic outline of the telodendrimer can be described as an architectural polymer (Figure 1), with one block being a linear polymer and the other a hyperbranched dendron. As mentioned above, much of the chemistry of the individual blocks has been the focus of investigations for many years, and is well described in the literature. Synthetic linear polymers can be dated back to the 19th century [11], and the first paper on the synthesis of dendrimers was published by Vögtle in 1978 [12]. Since then, a broad range of linear block-copolymers and dendrimers have been developed. The beginnings of polymer synthesis and functionalization of polymers in general can be traced to the vulcanization of natural rubber in 1839 by Charles Goodyear, and the preparation of nitrocellulose by Christian F. Schönbein in 1846. In 1907, the first fully synthetic polymer (Bakelite) was invented by Leo H. Baekeland, and it was prepared from the condensation of formaldehyde and phenol. In 1922, work by Staudinger lead to the understanding that polymers are in fact big molecules composed of atoms held together by covalent bonds [11]. Prior to that, it was believed that these are large aggregates assembled by non-covalent interactions between small molecules. In 1953, Staudinger received the Nobel Prize for his contribution to the understanding of macromolecular chemistry. Polymer chemistry continues to be the focus of intensive investigations, and has led to several novel discoveries, with more recent examples leading to applications in, for example, bullet-proof vests and 3-D printing. As the individual blocks in telodendrimers have been widely investigated, it is thus imperative that an introduction to each is provided first, before embarking on the detailed analyses of telodendrimers in regard to their synthesis, structural evaluation, and applications in biology.

## 2. Dendrimers/Dendrons

Globular hyperbranched macromolecules with well-defined and monodisperse tree-like architecture have been extensively investigated, since the first report by Vögtle in 1978 [12]. This divergent build-up was subsequently optimized using H_2_/Raney catalytic reduction by Meijer et al. [13], and Wörner and Mülhaupt [14] in 1993. Since the discovery of PPI dendrimers, a broad range of such structures have been synthesized, using a variety of chemical methodologies. Some of the dendrimers that may now be classified as “classic” include (i) poly(amidoamine) (PAMAM), which were developed by Tomalia in the 1980s, through an iterative reaction sequence between ethylenediamine and methyl ester moieties, and Michael addition with methyl acrylate on amines, in a divergent methodology [15,16]; and (ii) poly(ether), Fréchet dendrimers, prepared from 3,5-dihydroxy-benzyl bromide [17], in a convergent build-up on this core. Subsequently, several other interesting backbones of dendrimers/dendrons have been added [18,19,20,21,22,23,24,25,26,27], and include some earlier reports on silicon-containing dendrimers by Hadjichristidis [28]; polylysine by Denkewalter [29]; polyester by Newkome [30] and Ihre et al. [31]; phosphorus-containing dendrimers by Caminade and Majoral [32,33,34]; polyether dendrimer via OsO_4_ catalyzed oxidation of carbon-carbon double bonds [35]; peptide dendrimers [36]; amphiphilic PAMAM dendrimer with one dendritic block functionalized with sugar and the other with *N*-phthalic amide moieties [37]; and dendrimers for one-pot post bis-functionalization [38].

A chemical methodology that has found an extensive utility recently in the synthesis of dendrimers is the Cu(I)-catalyzed reaction between an alkyne and azide, leading to the formation of a 1,2,3-triazole moiety. This was introduced to the dendrimer field by Sharpless in 2004 [39], and later developed into a variety of dendritic architectures, including the synthesis of asymmetric PAMAM [40] and PAMAM/Fréchet [41] dendrimers; multifunctional dendrimers with PEG and fluorescent dye (BODIPY) [42,43]; dendrimers/nanocarriers by covalent attachment of dendrons on a PEG with azide moiety at both ends [44]; and dendrons linked to oligonucleotides [45]. It has also contributed to the post-functionalization of dendrimers, such as the conjugation of minocycline to a PAMAM dendrimer surface [46], dendrimers conjugated with bile acid [47], PEGylated PAMAM dendrimer covalently linked with camptothecin [48], a low-generation polyaromatic dendrimer containing oligosaccharides [49], as well as PAMAM dendrimers with DNA strands on the surface [50]. Malkoch’s group has reported the synthesis of bis-MPA dendrimers, and functionalized them with a range of moieties, demonstrating the high fidelity and efficiency of the reaction [51,52].

One limitation of the classic “click” reaction is the presence of copper that in many cases must be removed from the product due to its toxicity. It has led to the development of metal-free alkyne-azide coupling, such as strain promoted azide-alkyne cycloaddition (SPAAC) [53,54]. There are now several versions of this methodology that have been applied to the synthesis of dendrimers [55], and functionalization of dendrimers/dendrons with PEG [56] and MTX [57]. Alternatives to using a cyclooctyne in the copper-free azide-alkyne cycloaddition have also been reported [58,59].

In most reports, dendrimers are functionalized on the surface with the desired moieties, but the stoichiometry and the synthetic strategies make it possible to functionalize the interior of the dendrimer also with a high level of control. An example of this relates to the conjugation of a molecule of rhodamine B chromophore to a PAMAM dendrimer center, in order to investigate photo physical applications [60], and biological tracing in living cells [61]. Non-covalent entrapment of molecules in dendrimers has also been reported [62,63], including physical encapsulation of fluorouracil in a PEGylated PAMAM dendrimer [62].

The stepwise build-up of dendrimers, which requires purification/deprotection at each step, is in contrast to the polymerization employed for the synthesis of hyperbranched polymers, which involves less control and results in higher polydispersity. A few examples of the synthesis of hyperbranched polymers with polydispersity close to one have been reported. This includes ring-opening polymerization of glycidol, yielding hyperbranched poly(glycerol)s of molecular weights of up to 700,000 g/mol, and a PDI of 1.1–1.4 [64]. In general, the syntheses of dendrimers and dendrons are conducted in a step-wise manner, resulting in a layered structure (wherein each layer is referred to as a generation), and with a much better chemical, structural, and stoichiometric control. This is important, especially in investigations where structure–function relationships are the main focus.

Dendrimers continue to be the subject of a very broad range of investigations, with applications ranging from drug delivery [65,66,67,68,69,70,71], catalysis [72,73], hydrogels [74], fingerprint detection [75], and optical data storage [76]. The structural diversity of dendrimers is indeed broad, not only in terms of the dendrimer skeleton but also in terms of functionalization with the chemical conjugates, and post synthesis manipulations, which broadens the scope of dendrimers and dendrons for varied utility [77,78,79,80,81,82,83,84,85,86,87,88,89,90,91,92,93,94,95,96,97,98]. Dendron is the term used for a smaller section of the dendrimer, with a similar build-up, containing a focal point and monodisperse hyperbranched backbone.

**Figure 2:**
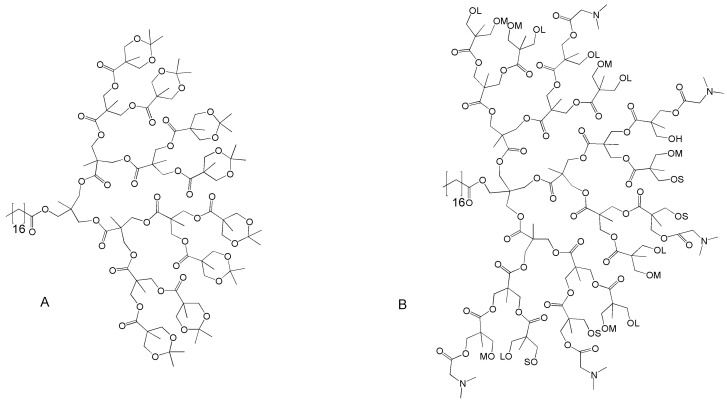
Polyester-based macromolecules reported by Alfei’s group: (**A**) [99]; in (**B**) (suffix M in the terminal moieties = l-arginine; L = l-lysine, S = sarcosine) [103].

One of the very interesting recent additions to the area of dendrimers is the polyester-based hyperbranched macromolecules, reported by Alfei’s group [99,100,101,102]. This elegant and versatile set of macromolecules (Figure 2) are intriguing alternatives to heavily utilized PAMAM dendrimers, with ease of tunable surface functionalization with a variety of functional groups, leading to neutral and charged nanostructures with low toxicities. These biocompatible scaffolds are promising candidates for applications in biology, as demonstrated by their potential in a series of therapeutic evaluations [99,100,101,102,103,104,105]. Such a structural outline, i.e., a dendron with an alkyl chain at the focal point, was previously reported by Newkome et al. [30], and investigated for its ability to form micelle structures.

## 3. Linear Polymers

**Scheme 1:**
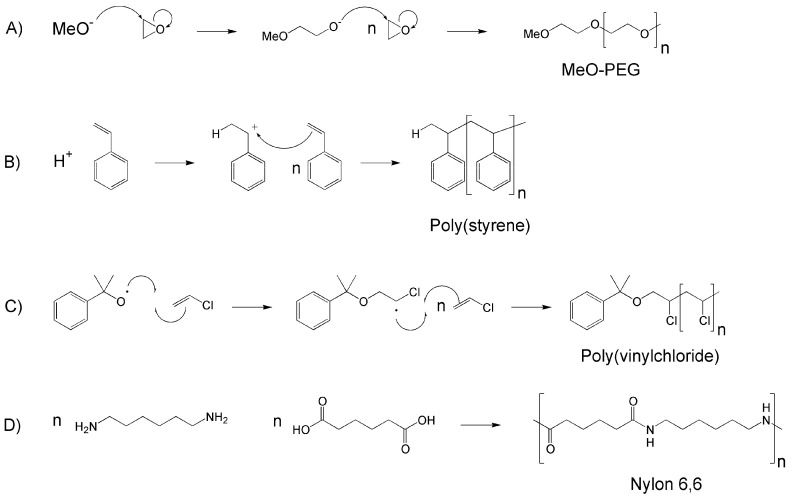
Different chemical reactions employed in polymer synthesis: (**A**) Anionic polymerization of epoxide with methoxide as anionic initiator; (**B**) Cationic polymerization of styrene with a strong protic acid as the initiator; (**C**) Radical polymerization of vinyl chloride initiated with a peroxide, and (**D**) Polycondensation of adipic acid and 1,6-hexanediamine to form the polyamide Nylon-6,6.

In the discussion of telodendrimers, which are rightfully described as hybrid structures, the other important constituent to consider is the linear polymeric architecture. One-dimensional polymers, such as poly(vinyl chloride) (PVC), poly(propylene), poly(amide), poly(ester), poly(styrene) (PS), poly(ethylene glycol) (PEG) etc., have found use in a wide range of applications, including tubing, kitchen appliances, textiles, insulation (thermic and electronic), and drug delivery [11]. In contrast to dendrimers, linear polymers, in general, have PDIs that are often higher than those for dendrimers and dendrons. There are several different methodologies that have been employed to synthesize linear polymers, including anionic, cationic, and radical polymerization [4,5]. For example, (i) PEG is typically prepared from the anionic ring-opening polymerization of epoxides (Scheme 1A); (ii) poly(vinyl alcohol) from vinyl acetate, followed by hydrolysis of the ester moieties of the resulting poly(vinyl acetate); (iii) poly(esters), by ring opening reaction of lactones; and (iv) poly(amides) by the condensation of diamines and diesters. A few examples of these methodologies are illustrated in Scheme 1.

The synthesis of polymers has expanded to include poly(peptides) and poly-nucleic acids, by taking advantage of the broad selection of coupling reagents and techniques that have become available [11]. Block-copolymers were introduced in the early 1950s, and it allowed the synthesis of polymers in which different monomers could be incorporated in blocks or alternating along the linear polymer chain. A variety of methodologies have been developed, including the use of living polymerization, that have allowed the syntheses of linear copolymers with varied backbones of their different blocks [4,5]. The applications of linear polymers in pharmaceutics have been the focus of intense investigations in the past few decades, and one of the early examples was the use of PEG in 1977 [106]. It was reported that catalase modified with PEG showed enhanced circulation times in the blood stream of mice. The enzyme retained 93–95% of the reactivity, and there was no evidence of an immune response post-injection. Since then, the incorporation of PEG has become one of the most important approaches to improve the pharmacokinetics of a wide range of drug molecules, including the use of PEG for the targeted and reductive sensitive delivery of DOX [107], as well as PEGylation of virus vectors [108] and dendrimers [109], demonstrating the versatility of this polymer.

PEG is commercially available in a wide range of molecular weights (M_w_) with low PDI, and with α and α/ω functionalities, such as alkyne, acrylate, amine, azide, biotin, bromo, NHS ester, and thiol. The use of PEG is, however, not without concern as reviewed recently [110,111,112], and some of the major issues in employing PEG in biomedical applications include bioaccumulation, as PEG is not biodegradable; immunogenicity, which may lead to accelerated blood clearance; and general toxicity. In an in vitro case study on Doxil [113], it was found that antibodies contributed to the activation, which meant the body may adapt to PEG as an unwanted foreign compound. In another study, the rapid clearance of PEG-asparaginase was found to be associated with the presence of antibodies against PEG [114]. 

Other linear polymers that have been used in relation to drug delivery include poly(vinyl alcohol) [115], poly(amino acids) [116], poly(*N*-(2-hydroxypropyl) methacrylamide) [117], and poly(*N*-vinyl-2-pyrrolidone) [118]. In addition, a few polysaccharides have also been under investigation, including chitosan [119], dextran [120,121,122,123], and hyaluronic acid [124]. Polymers from *N*-(2-hydroxypropyl) methacrylamide have been studied as water-soluble delivery systems for anticancer drugs [117], but as was the case with PEG, such polymers are not biodegradable. However, some strategies to overcome this limitation have also been reported. For example, these polymers can be made biodegradable by the incorporation of desired moieties that will break down, using cues in a biological environment. In a study of this kind in 2011 [125], enzymatically degradable oligopeptide sequences were incorporated in a multiblock *N*-(2-hydroxypropyl) methacrylamide copolymer, which made the multiblock copolymer biodegradable. Amphiphilic linear block copolymers have also been used in the delivery of several hydrophobic drugs [122,126].

Linear amphiphilic block-copolymers have offered an interesting platform to design a variety of self-assembled structures for physical encapsulation of active pharmaceutics [127,128,129]. Through articulation of their hydrophobic and hydrophilic components, one can obtain aqueous self-assemblies with morphologies including micelles, polymersomes, inverted micelles, etc. [130,131]. There is tremendous diversity in the chemical compositions of the blocks in such polymers, which has evolved over the years, and have helped tailor specific formulations with intended tasks [127,128,129,130,131,132,133]. One could imagine a similar variable composition included into the blocks that constitute telodendrimers, considering the range of dendrimers and linear polymers that are now available. Bringing a similar variation in amphiphilicity through the linear block and hyperbranched dendron, the resulting self-assemblies could be tailored to desired needs and with required features. Considering that micellar formulations are the most extensively studied, it is possible to tune the core/corona density in architectural polymers, and use it to one’s advantage in drug delivery. A detailed evaluation of such assemblies is necessary through variations in the linear and hyperbranched blocks, and this is an area where we expect to see tremendous growth in coming years. One of the constraints at this stage is the limited number of groups working in this area, and most studies are restricted to a handful of dendrons, and polyethylene glycol being the major linear polymeric arm. It is understandable considering their applications in biology, but we also hope that researchers will start to consider other hydrophilic polymeric arms, and bring the diversity of block-copolymers into telodendrimers.

## 4. Telodendrimers

**Figure 3:**
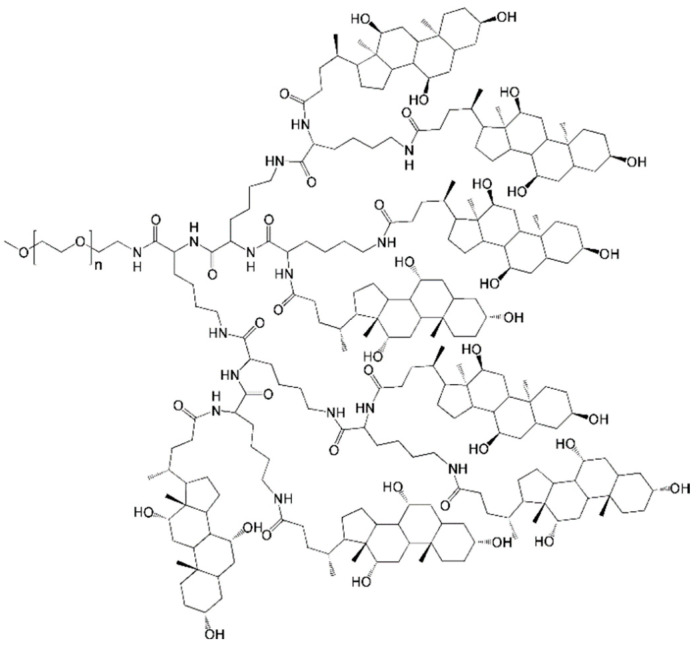
The basic outline of a PEG-poly(lysine) telodendrimer functionalized with cholic acid on both the α and ε amines of the lysine moieties.

As shown in Figure 1, the basic architectural design of telodendrimers may be described as a diblock copolymer AB, consisting of a linear polymer (A), such as PEG, a poly(ester), or a poly(amide); and a hyperbranched dendron, including PAMAM-, polyester-, and polyamine (B). The majority of the published examples of telodendrimers are composed of a hydrophilic linear polymer, such as PEG, coupled with dendrons, such as poly(lysine) or poly(ester), functionalized with hydrophobic moieties. Figure 3 shows such a telodendrimer containing a PEG-poly(lysine) backbone, functionalized with cholic acid on the α and ε amines of the lysine moieties.

**Scheme 2:**
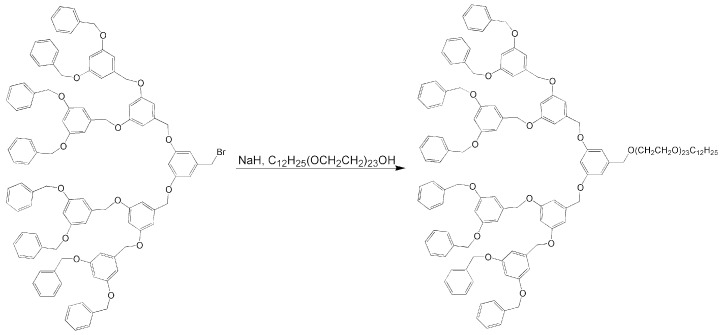
Coupling of a dendritic block with a PEG block [135].

**Scheme 3:**
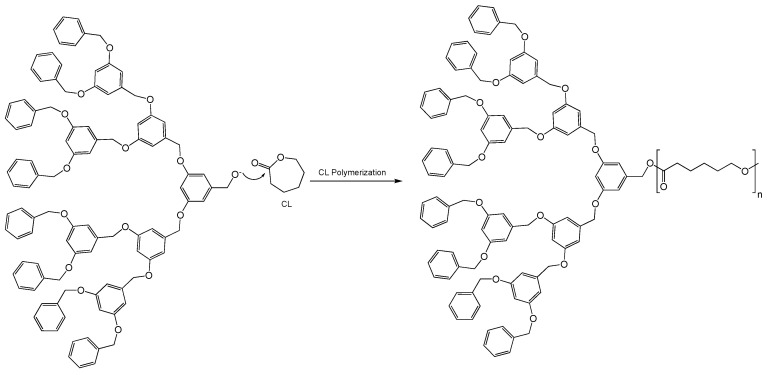
Using dendron as a macroinitiator, ring-opening polymerization of ε-caprolactone (CL) [138].

**Scheme 4:**
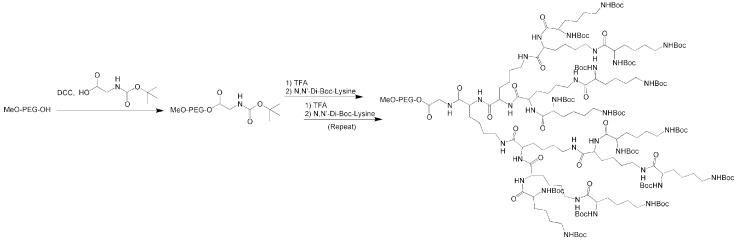
Synthesis of poly(lysine) dendron using the terminus of the PEG as the starting point [140].

In general, there are three strategies that have been adapted for the synthesis of telodendrimers: (i) The dendrons are built from a commercially available or pre-made linear polymer; (ii) the polymerization of the linear block is initiated from the focal point of the dendron; and iii) a dendron is covalently linked to a linear polymer. These strategies are illustrated in Scheme 2, Scheme 3 and Scheme 4 (vide infra) with examples from the literature. The chemical reactions and methodologies that have been developed for the individual blocks of a telodendrimer are very relevant in planning their syntheses. The same is true for the structural considerations when designing new architectural polymers with desired physical, chemical, and biological characteristics. As telodendrimers are composed of both linear and dendritic polymer blocks, they share physical, biological, and chemical similarities, to some extent, with both components. This suggests that insights developed from investigations on the individual polymers can be useful in the study of telodendrimers, in theory. Hybridization of these two types of polymers combines the properties of the individual polymer type, and offers many architectural opportunities for a variety of applications. As per the scope of this review, the applications are, however, limited to biology.

The architectural design of the telodendrimer may be dated back to the mid-1980s, when, in search of new micelle structures, Newkome’s group designed and synthesized molecules composed of linear alkyl chains as the hydrophobic block, and dendritic polyester or polyamide with hydroxyl moieties as the hydrophilic block [30]. This study described preliminary methods for the design and synthesis of an amphiphilic architecture, which later became known as telodendrimers. In the early 1990s, work on telodendrimers began to appear, and since the syntheses of individual blocks (dendron and the linear polymer) were already well developed, the focus of the chemical investigations was now more aimed at (i) how to join the blocks to yield the desired hybrid structure, and (ii) investigate whether it was possible to grow a polymer from the sterically hindered center of a dendron, or grow a dendron from the terminus of a linear polymer. For example, a Fréchet-type dendron with benzyl alcohol at the focal point was reacted with *p*-chloromethyl styrene, which was subsequently polymerized through free-radical polymerization to build the hydrophobic block from the focal point. Different generations of the dendron were introduced, and the feed rate of styrene was varied to develop a variety of such architectural polymers [134].

Another telodendrimer with a Fréchet-type dendron was published in 1992, where a bromo methyl moiety at the focal point of the dendron was reacted with a deprotonated alcohol moiety at the terminus of PEG, in a Williamson-type ether synthesis (Scheme 2) [135]. Such telodendrimers were used to investigate physical properties, including aqueous self-assembly, leading to micelle formation [136]. The Fréchet dendron-PEG system could also be constructed easily by coupling a dendron on to the PEG terminus. A transesterification reaction between a methyl ester at the dendron focal point and the PEG hydroxyl terminus has also been used to link the two structures [137].

Using the Fréchet-type dendron as macroinitiators, the kinetics of the ring-opening polymerization of ε-caprolactone was analyzed (Scheme 3), and the anion used for the polymerization initiator at the focal point of the dendron was in the form of a deprotonated benzylic alcohol [138]. With this strategy, it was possible to synthesize a Fréchet dendron-polyester diblock copolymer, and the polymerization could be put under kinetic control. 

The deprotonation of benzylic alcohol at the focal point of a Fréchet-type dendron has also been used to initiate the anionic polymerization of ethylene oxide, resulting in a narrow molecular weight distribution of PEO (M_w_/M_n_ = 1.02–1.04) [139]. After the polymerization reaction, the living polymer anion was treated with either HCl/THF, or a reactive dendron, to synthesize di- or tri-block copolymers, respectively.

The synthesis of a telodendrimer with a poly(lysine) dendritic block was reported in 1994, where the hydroxyl moiety on mono-hydroxy PEO was reacted with *N*-Boc-l-Glycine, upon which, after deprotection, a poly(lysine) dendron was built divergently (Scheme 4). The terminal amines on the dendron periphery were decorated with *^t^*Bu-carbamate [140].

A polystyrene-poly(propylene imine) telodendrimer was prepared starting from an amine-terminated polystyrene block. The dendron was constructed in a classic iterative fashion (divergent methodology), with acrylonitrile, in a conjugate addition to amine, followed by reduction of the nitrile to the primary amine. This strategy was similar to the one described for the synthesis of one of the first examples of dendrimers by Vögtle [12], Meijer [13], and Wörner and Mülhaupt [14]. The self-assembly behavior of these polymers, leading to the formation of micelles, was investigated, and it was found to be generation dependent [141,142].

**Scheme 5:**
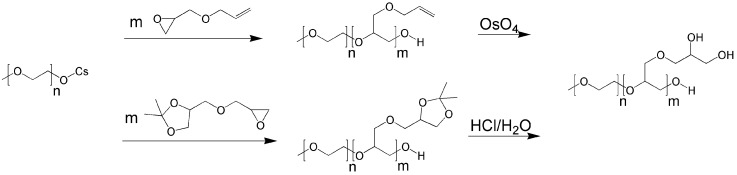
Synthetic strategies for PEG-poly(glycerol) pseudo-telodendrimer.

Two strategies for the synthesis of PEG coupled with polyether and (poly(glycerol)) dendrons have been reported [143], and in both cases, hyperbranched blocks were synthesized onto prefabricated PEG. The polydispersity index was found to be narrow (1.05–1.18). In another example, similar to the methodology introduced by Haag [35] in the synthesis of poly(ether) dendrimer, an allyl ether double bond was oxidized using OsO_4_ as a catalyst to introduce two geminal hydroxy moieties. The drawback of this strategy is the use of OsO_4_, as every trace of osmium must be removed if such compounds are to be used in biology. This disadvantage was overcome by building a pseudo dendritic block, which is composed of an epoxide unit for constructing PEG, and an acetal-protected glycerol (Scheme 5). Using a similar approach, carbosilane chemistry has been used to prepare a poly(carbosilane) dendritic block, leading to PEG-poly(carbosilane) telodendrimer-type architectures [144].

The focus of these early investigations was the chemistry and structural properties, and no biological evaluations were reported. However, these studies did introduce the basic structural outline and strategies for the synthesis of telodendrimers, in which the overall architecture could be tuned with variations in the hydrophobic and hydrophilic blocks. These results demonstrated that it is indeed possible to combine the linear polymers with dendrons, and with the structural diversity available for both, it was envisioned that the combination of these two will open the doors for a broad range of applications. As described later, the basic architectural outline and the synthetic strategies used to build telodendrimers have expanded due to an interest in more advanced structures with multiple functionalities.

As the general chemical methodologies involved in the synthesis of telodendrimers were becoming available, the biological perspective of their applications became the next focus [145,146,147,148,149,150,151,152,153,154,155,156,157,158,159,160,161]. As noted by Newkome in 1985, it was the search for an architectural outline with the ability to form micelles that inspired the scientific community about these hybrid structures. Micelle formation still plays the central role in studies conducted to this day, and telodendrimers have been used in developing nanocarriers for the delivery of a variety of drugs. Most of these studies are indeed related to cancer and/or diagnostics. Targeted delivery of chemotherapeutic drugs to tumors is still a major challenge, and the development of efficient drug delivery technologies continues to be one of the important goals in soft nanoparticle research. Anticancer drugs are by virtue toxic, as they are designed to kill malignant cells, and these must be guided to the diseased cells or tissue to avoid the attack on healthy counterparts. These active agents are also generally lipophilic, and poorly soluble in a biological medium. The hydrophobic nature of these drugs is important for their interaction with the targeted cellular receptors, and introducing moieties to render hydrophilicity generally reduces their efficiency. One of the anticipated purposes of drug delivery systems, including nanocarriers from telodendrimers, is to improve the pharmacodynamics and pharmacokinetics of these drugs, as well as facilitate their accumulation at disease sites.

Factors, such as their ability to form self-assembled structures in an aqueous medium, critical micelle concentration (CMC), micelle size, loading capacity, circulation time, tumor accumulation, in addition to their toxicity, are important factors to consider in developing telodendrimer-based nanocarriers. A telodendrimer composed of PEG-lysine dendron was used to form micelles [162] of 20–60 nm diameter, which had a drug loading capacity of about 7.3 mg PTX/mL. The nanocarrier accumulated in ovarian tumors, and showed an enhanced therapeutic efficacy compared to Taxol^®^ or Abraxane^®^. Subsequently, a series of telodendrimers of the PEG-poly(lysine)-cholic acid structure were prepared, in which the length of PEG, and the number of cholic acid moieties were varied. Using in vivo studies, it was demonstrated that the telodendrimer-based micelles with diameters ranging from 17–64 nm from these telodendrimers were more efficiently accumulated at tumor sites, compared to micelles of 154 nm, which were found to have a very high uptake in the lung and liver [163].

Telodendrimers composed of poly(*ε*-caprolactone) of 3.5 or 14 kDa linear blocks, and a generation 3 poly(ester) dendritic block, prepared from 2,2-bis-(hydroxy-methyl) propionic acid, with PEG chains of 2 or 5 kDa on the surface, have been prepared. The linear polymer block had an azide moiety at its terminus, and the dendron with an alkyne at the focal point, and the two were linked together through high-yielding alkyne-azide click reaction. For comparison, linear diblock copolymers of composition poly(ε-caprolactone)-PEG were also prepared, and it was found that the CMC of the telodendrimer-based assemblies, in general, was much lower than those from the linear block co-polymers. The lowest CMC for all the derivatives was 0.65 × 10^−7^ M for the PCL_14kDa_-G3-PEG_2kDa_ telodendrimer [164].

In another example of telodendrimer-based self-assemblies with low CMC [165], a poly(ester)-type dendritic block, prepared from 2,2-bis(hydroxy methyl)propionic acid, was used to build a poly(γ-n-dodecyl-l-glutamate) linear hydrophobic chain. The dendron was PEGylated with units containing terminal carboxylic acids. The CMC of the resulting micelles from this telodendrimer was found to be of the order of 10^−8^ M, and the particle size was pH dependent, due to acidic moieties on the surface of the micelles. Using the bactericide triclosan as the encapsulated agent, a loading capacity of 30 wt% was observed. The authors speculated that the micelles can be functionalized with bio-specific ligands, and the system can be used for targeted drug delivery applications.

**Figure 4:**
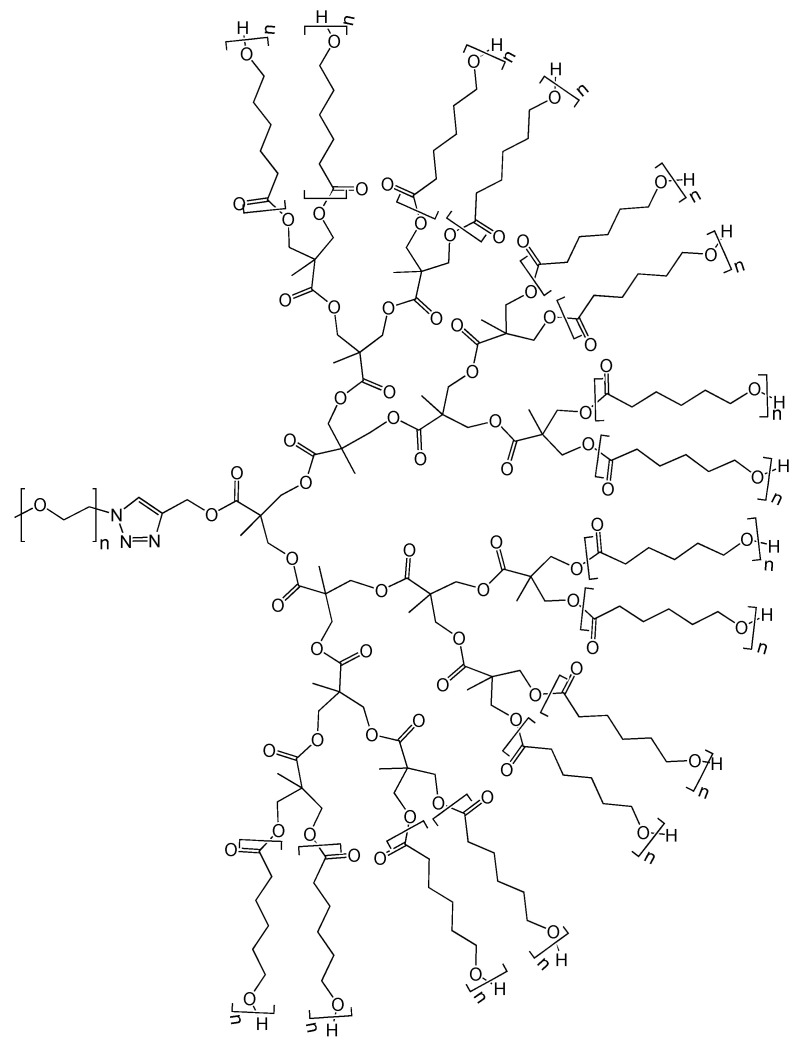
2,2-bis-(hydroxy-methyl) propionic acid-based dendrimer, functionalized with poly(ε-caprolactone) chains at the termini, and PEG at the core [166].

A series of telodendrimers with a linear PEG and a dendritic block of different generations composed of 2,2-bis-(hydroxy-methyl) propionic acid, functionalized with poly(ε-caprolactone) chains, was synthesized and investigated for potential drug delivery applications, as shown in Figure 4 [166]. The alkyne-azide and thiol-ene click reactions were used to couple PEG to the focal point of the dendron. The thiol-ene coupling had a shorter reaction time and did not require a metal catalyst, while the alkyne-azide reaction required an excess of PEG. It was reported that the generation of the dendron had a profound effect on the amount of DOX that could be encapsulated.

Self-assembly of a series of PEG-poly(lysine)-based telodendrimers, containing a number of camptothecin moieties on the dendritic block, were shown to form nanospheres or nanorods, depending on the structure of the telodendrimers. Their in vitro and in vivo behavior was strongly affected by the shape and size of these assemblies, as nanorods smaller than 500 nm had longer blood circulation and faster cellular uptake than the nanospheres and larger nanorods [167].

**Figure 5:**
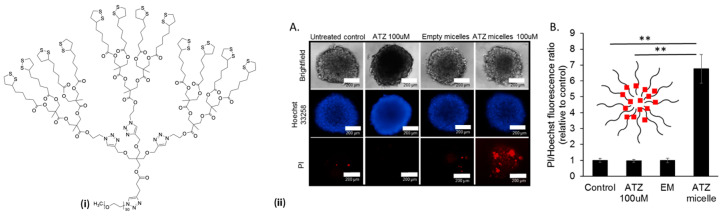
(**i**) Telodendrimer for combination therapy. (**ii**) Viability of glioblastoma spheroids treated with ATZ: (**A**) U251 spheroids treated with ATZ, empty telodendrimer micelles, and ATZ-loaded micelles; (**B**) Relative PI to Hoechst 33342 fluorescence intensity. ** indicates statistically significant differences. Reprinted with permission from [168]. Copyright 2017 American Chemical Society.

A versatile and easily adaptable synthetic strategy was developed by Choi et al. [168], in which the telodendrimer was prepared using the alkyne-azide click reaction, both in the synthesis of the dendritic block, and in the coupling of the dendritic block with commercially available linear PEG. The dendrimer was based on a poly(ester) backbone, prepared from 2,2-bis(hydroxy methyl) propionic acid, and a derivative of this telodendrimer was functionalized on the dendritic surface with lipoic acid (Figure 5). It was self-assembled into micelles, and loaded with active agents for combination therapy. It was observed that the cargo loading efficiency was drug specific, and was found to be low for quercetin, while high for acetazolamide. The acetazolamide-loaded micelles significantly reduced GBM cell viability in 3-D spheroids. These results suggest that achieving high loading of drugs into nanocarriers requires a good fit between the drug and the telodendrimer core composition.

### 4.1. Telodendrimers with Reverse Hydrophobic/Hydrophilic Blocks

Not all telodendrimers that have been investigated for drug delivery have the composition of a hydrophilic linear block and a hydrophobic dendron. An interesting example of the opposite was published in 2010, in which the linear hydrophobic block consisted of poly(benzyl-l-aspartate), and the dendrimer had a polyester backbone with short PEG chains attached at the periphery [169]. By mixing folate-functionalized telodendrimers with unfunctionalized analogs in different ratios (0% to 100%), blends could be obtained, in which the total amount of folate moieties was the same. KB cells overexpressing folate receptors were used to evaluate the binding of the micelles, and it was reported that when using telodendrimers with 20% folate and 40% unfunctionalized telodendrimers, one could obtain the highest cell association, after 24 h of incubation. This was also confirmed by in vivo experiments, and the studies showed that fine tuning of ligand cluster arrangements is important in optimizing the targeting of micelles.

### 4.2. Telodendrimers in Protein Delivery and Ligand—Receptor Interactions

**Figure 6:**
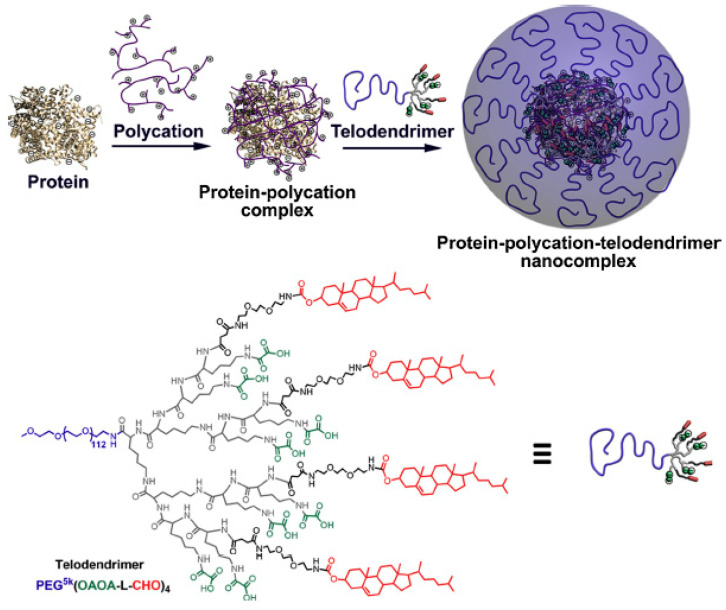
Telodendrimer and the methodology to construct protein-polycation-telodendrimer nanocomplexes. Reprinted with permission from [171]. Copyright 2018 Elsevier.

A multifunctional telodendrimer of composition, PEG-poly(lysine), was synthesized using selective couplings, where an amine of lysine moieties was reacted with (Fmoc)Lys(Fmoc)-OH, followed by the reaction of the other with, for example, Fmoc-Arg(Pbf)-OH [170]. Using this strategy, a library of telodendrimers with different hydrophobic (heptadecanoic acid, cholesterol, or d-α-tocopherol) and charged moieties (arginine, arginine_2_, lysine_2_, or oxalic acid_2_) were synthesized. The hydrophobic groups were conjugated to the dendron through a PEG_5kDa_ spacer. These telodendrimers were used to optimize protein loading, and it was found that the ionic strength, hydrophobicity, and the combinations of these two and their densities were important for efficient protein encapsulation. Using a similar approach, a telodendrimer functionalized with oxalic acid and cholesterol, was evaluated for the encapsulation of protein-polycation complexes (Figure 6) [171]. This strategy overcame limitations encountered using polycationic vectors for intracellular delivery of proteins, such as aggregation and non-specific interactions to other biomolecules.

The binding of ligands to receptors has also been examined using a telodendrimer, where PEG with an amine terminus was reacted with a gallic acid derivative, in which three hydroxyl moieties had been alkylated with triethylene glycolazide, yielding the first-generation azide-terminated architectural polymer. Further generations were prepared by reduction of the azide to amine using catalytic hydrogenation, and then sequentially coupled to the gallic acid derivative. The azide moieties were then used to decorate the dendrons in a click reaction with alkyne-functionalized carbohydrates (α-d-mannose, α-l-fucose, and β-d-lactose). It was reported that higher generation dendrons had elevated capacities to aggregate lectins, suggesting that such architectures could be used to study carbohydrate–receptor interactions in future investigations [172].

### 4.3. Passive Targeting: Accumulation Using the EPR Effect

Telodendrimer-based assemblies are generally designed to take advantage of the enhanced permeation and retention (EPR) effect to target tumor cells, as with other polymeric soft nanoparticles [173]. The EPR effect is a consequence of the tumor vasculature displaying an increased permeability and limited lymphatic drainage. A variety of structurally diverse telodendrimers have been investigated to understand their ability to deliver drugs using EPR. For example, a series of telodendrimers based on PEGs of different lengths, and lysine and cholic acid-based dendrons were prepared by varying the subunits of telodendrimers, so that the physiochemical properties, such as particle size, CMC, and drug loading capacity, could be fine-tuned. It was observed that high levels of hydrophobic drugs, such as PTX and SN-38, could be loaded into micelles formed from these telodendrimers, with a loading capacity of 37.5% *w/w* for PTX, for example. Using infrared fluorescence imaging on mice with ovarian cancer xenograft, it was shown that the PTX-loaded micelles showed better antitumor efficacy when compared to Taxol^®^, at equivalent doses [174].

PEG-poly(lysine) telodendrimers decorated on the dendritic block with cholic acid moieties (PEG_5k_-CA_8_ and PEG_2k_-CA_4_) were used to investigate DOX-loaded micelles. The loading capacity was found to be 14.8% for PEG_2k_-CA_4_ and 8.2% for PEG_5k_-CA_8_ (*w/w*), and both showed prolonged blood circulation, and preferentially accumulated in B-cell lymphomas, compared to free DOX. The maximum tolerated dose for the PEG_2k_-CA_4_ derivative loaded with DOX was 1.5-fold higher than free DOX [175]. This study highlights the significance of tuning the telodendrimer structure to confer specific properties.

**Figure 7:**
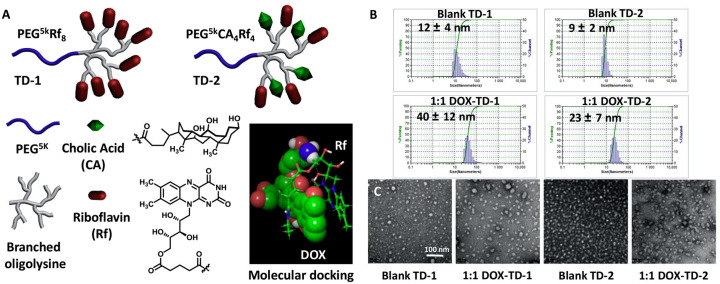
PEG-polylysine dendrimers (**A**), nanoparticle sizes (**B**), and TEM images (**C**) of empty and DOX-loaded assemblies. Reprinted with permission from [177]. Copyright 2017 Elsevier.

Four telodendrimers based on PEG-poly(ester)/poly(amide) decorated with farnesylthiosalicylate (FTS), and with compositions of PEG_2k_-FTS_2_, PEG_2k_-FTS_4_, PEG_5k_-FTS_2_, and PEG_5k_-FTS_4_, were synthesized and self-assembled into micelles. Their CMC, drug loading capacities and ability to deliver PTX to tumor cells in vitro as well as in vivo were evaluated, and the PEG_5k_-FTS_4_ micelles with PTX were most effective in inhibiting tumor cells in vivo [176]. In another study, telodendrimers based on PEG and poly(lysine) were synthesized and tested for their ability to deliver DOX. One of these was functionalized with riboflavin, and the other with riboflavin and cholic acid moieties on the dendritic block. High loading capacities, prolonged circulation time in blood plasma, and efficient tumor targeting were observed, together with antitumor efficacy (Figure 7) [177].

The above studies demonstrate that targeting using the EPR effect is a promising strategy, and the diversity in telodendrimer compositions, which allows changing parts of the carrier system, affects important properties, such as CMC and drug loading capacities.

### 4.4. Active Targeting Using Functional Moieties on the Telodendrimer

Besides taking advantage of the EPR effect, a few studies on a more active targeting strategy have been carried out by the functionalization of telodendrimers with peptides as ligands. For example, telodendrimers were covalently functionalized with a targeting peptide for the delivery of daunorubicin. The micelles could load 5 mg of daunorubicin per 20 mg of telodendrimer, and the micelles could transport the drug into the cells expressing the C-type lectin, and to leukemia stem cells in vitro. The nanoparticles did not bind to normal blood cells or to normal hematopoietic stem cells, demonstrating the selectivity of this system [178].

A PEG-poly(lysine)-cholic acid telodendrimer with a targeting peptide (OA02) that had a high affinity for the α-3 integrin receptor, overexpressed on the surface of ovarian cancer cells, was synthesized. The alkyne-azide click reaction was used to functionalize the telodendrimer with the peptide, and it was found that this functionalization did not have much of an effect on its physicochemical properties, but the uptake was significantly improved in SKOV-3 and ES-2 ovarian cancer cells via receptor-mediated endocytosis. Upon loading paclitaxel, the nanocarrier’s in vitro cytotoxicity was also improved. In vivo studies showed that PTX-loaded micelles had increased tumor and lower systemic toxicity in mice, compared with non-targeted micelles, as well as Taxol [179]. PEG-poly(lysine)-cholic acid telodendrimers with a targeting peptide at the PEG block were also used to investigate if micellar formulation of daunorubicin modifies the pharmacokinetics of the drug, in order to increase the drug exposure of leukemic cells, and reduce cardiac toxicity, compared to the free drug. The nanoformulation dramatically reduced the cardiac toxicity of daunorubicin, and increased the antitumor effect in vivo [180].

Telodendrimers based on PEG, in which one was decorated with eight cholic acid units at the dendritic block and a PLZ4 targeting entity (a cyclic peptide), and the other with four cholic acid and four pyropheophorbide A moieties, were synthesized. These telodendrimers were mixed together with DOX, in order to prepare drug-loaded micelles. The PLZ4 bound specifically to the αvβ3 integrin on bladder cancer cells, and the nanoparticles loaded with DOX selectively targeted tumor cells, and could serve as a triple-modality therapeutic agent against bladder cancers (i.e., photodynamic, photothermal, and chemo) [181]. The cyclic peptide PLZ4 has also been used to target bladder cancer [182], and these examples demonstrate the varied design possibilities of telodendrimers as drug carriers.

### 4.5. Telodendrimers in Controlled Release 

Besides the targeting ability of the drug carrier systems, the controlled delivery of the pay loads is also of great importance. Telodendrimers can be designed with the ability to respond to autogenous stimuli, such as pH, reduction, and enzymatic activity. A range of telodendrimers with different stimulus responsive units have been investigated, and these studies are summarized below.

#### 4.5.1. Disulfide Cross-Linking 

Telodendrimer-based micelles can be stabilized by cross-linking using disulfide bridges, and in most cases, the sulfurs are introduced as cysteine moieties, which can then be oxidized to form the disulfide bond to cross-link the telodendrimers in a micelle. With more stable micelles, the circulation times may increase, which in combination with the EPR effect may result in tumor accumulation and reduced systemic toxicity. In one such example, telodendrimers composed of a linear PEG, lysine as branching units, and Ebes (a hydrophilic spacer), cholic acid, as well as cysteine (for cross-linking) built into the dendritic block, were used to form micelles to transport PTX. This nanocarrier was compared to a non-cross-linked analog, and was found to have a higher loading capacity, enhanced micelle stability, and longer in vivo circulation times. The cross-linked micelle PTX formulation was found to be more efficient in an ovarian cancer xenograft mouse model, compared to the non-cross-linked version, and Taxol^®^ with an equivalent dose. The release could be further triggered by the administration of the reducing agent *N*-acetylcysteine [183]. A similar study on the delivery of DOX in the treatment of B-cell lymphoma was later published from the same group, demonstrating the versatility of their strategy [184]. By mixing telodendrimers with and without cysteine moieties, they investigated the optimal level of cross-linking in relation to micelle properties, such as stability, drug release, and responsiveness to the reductive environment. It was reported that cross-linking levels of 20% and 50% were more effective antitumor formulations against in vivo SKOV-3 ovarian cancer cells [185].

Mixing two different telodendrimers to form micelles was the approach in another study, in which a telodendrimer containing cysteines for cross-linking via disulfide bond formation was mixed with the one that had indocyanine green with photothermic properties. Both telodendrimers were composed of PEG as a hydrophilic linear block, and poly(lysine) as the dendron skeleton decorated with cholic acid moieties. The cross-linked micelles with photothermal functionalities could also be loaded with DOX or imiquimod to target tumor sites [186].

**Figure 8:**
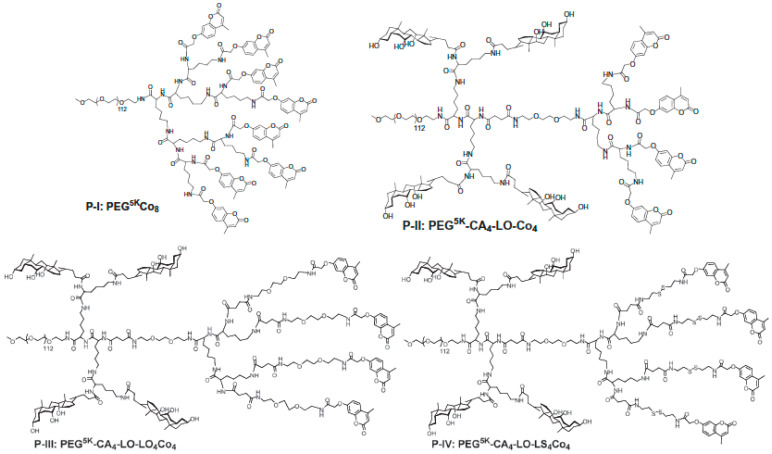
PEG-poly(lysine) telodendrimer with different backbones and a variety of functional groups. Reprinted with permission from [188]. Copyright 2015 Elsevier.

Another strategy to control nanoparticle stability used coumarin moieties built into the dendritic block, and photo-dimerized in order to cross-link the telodendrimers within micelles. The cross-linking leading to dimerization was carried out with light of the wavelength ≈ 310 nm, and the reverse reaction (i.e., removing the links) with a wavelength of about 254 nm. The macromolecular construct also contained disulfide moieties in the dendritic skeleton, for reduction-responsive drug release [187]. The redox and photo-responsiveness of disulfide and coumarin have also been combined in a PEG-poly(lysine) telodendrimer, which was decorated with cholic acid (Figure 8). The nanocarrier was investigated for the delivery of SN-38 in the treatment of colon cancer, and was found to accumulate in tumors, instead of the healthy tissue. The SN-38-loaded micelles exhibited higher antitumor efficacy, compared to the commonly used drug, irinotecan, in the treatment of colon cancer at equivalent doses in HT-29 human colon cancer xenograft models [188].

#### 4.5.2. pH-Responsive Nanocarriers

An example of pH-responsive telodendrimers was published in 2004, in which linear PEG chains were used for the convergent build-up of poly(ester) or poly(lysine) dendrons, functionalized with hydrophobic and hydrolysis-active acetals. These hybrids formed stable micelles in aqueous solutions at neutral pH, and disintegrated at pH 5, due to hydrolysis of the acetals, and triggering release of the encapsulated Nile Red [189]. pH-based release of the payload has also been utilized in the reversible formation of boronate esters from boronic acids and diols [190]. PEG-poly(lysine)-cholic acid telodendrimers were prepared, and functionalized with boronic acid and catechol, respectively. The release of PTX from such cross-linked micelles was significantly slower, compared to non-cross-linked analogs. The drug release could be accelerated at lower pH, and/or in the presence of mannitol, suggesting that the latter could be used as a trigger, together with the lower pH in the tumor microenvironment.

#### 4.5.3. Enzyme-Responsive Telodendrimer Assemblies 

**Scheme 6:**
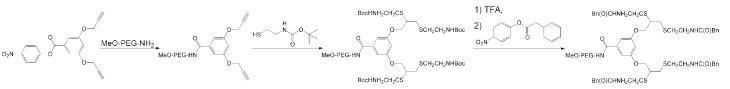
Synthesis of enzyme responsive telodendrimer.

Telodendrimer-based micelles responsive to enzymatic activity have been prepared using PEG as the linear hydrophilic block, and a dendron linked with hydrophobic phenyl acetamide moieties. The latter are responsive to penicillin G amidase, and could be cleaved using enzymatic activity [191]. A second-generation dendron was prepared by reacting 3,5-bis(prop-2-yn-1-yloxy) benzoic acid with four thiols, yielding two geminal thioethers. A series of telodendrimers containing PEG of 2, 5, or 10 kDa were synthesized (Scheme 6). A detailed evaluation of the resulting micelles upon self-assembly, and their disassembly in response to enzymatic activity using Nile Red suggested that the release rate can be tuned by varying the length of PEG.

### 4.6. Drugs for Combination Therapy

**Figure 9:**
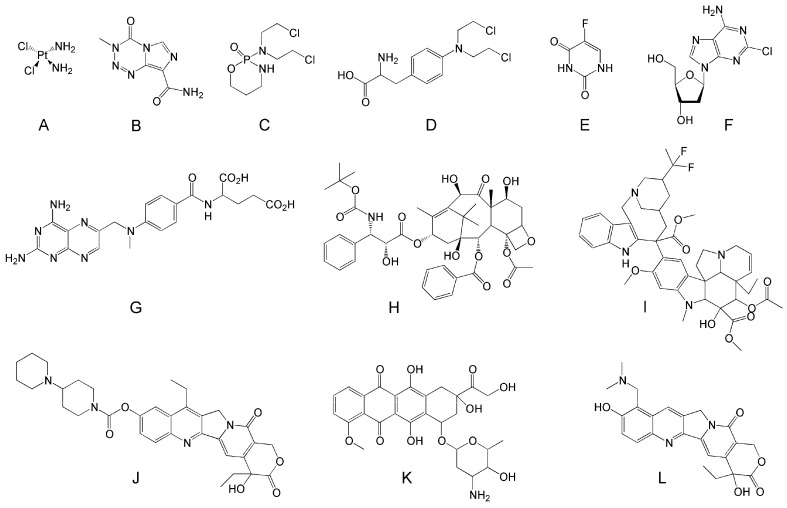
Chemical structures of anticancer drugs: (**A**) Cisplatin, (**B**) Temozolomide, (**C**) Cyclophosamide, (**D**) Melphalan, (**E**) Fluorouracil, (**F**) Cladribine, (**G**) Methotrexate, (**H**) Docetaxel, (**I**) Vinflunine, (**J**) Irinotecan, (**K**) Doxorubicin, and (**L**) Topotecan.

In order to reduce drug resistance in the treatment of high morbidity rate diseases, such as cancer, administration of two different pharmaceutics in a combination therapy is considered an advantageous approach. The purpose of most anticancer agents is to trigger apoptosis and kill malignant cells. This can be achieved in many different ways, and anticancer drugs can be grouped according to their mode of action. To glance at the structural diversity of anticancer drugs, and as the list is pretty large, a few representative examples are shown in Figure 9. Alkylating anticancer compounds make new covalent bonds on biologically important molecules, such as DNA, which may cause severe damage to the cell, leading to cell death. Examples of these include cisplatin (Figure 9, **A**), temozolomide (Figure 9, **B**), cyclophosamide (Figure 9, **C**), and melphalan (Figure 9, **D**). Fluorouracil (Figure 9, **E**), cladribine (Figure 9, **F**), and folic acid antagonists, such as methotrexate (Figure 9, **G**), are examples of antimetabolites used to block the incorporation of purines and pyrimidines in DNA and RNA. Antimitotic agents, such as docetaxel (Figure 9, **H**), paclitaxel, vinflunine (Figure 9, **I**), and vinorelbine, are drugs that bind to microtubules and inhibit mitosis. The topoisomerase inhibitors, such as irinotecan (Figure 9, **J**), doxorubicin (Figure 9, **K**), topotecan (Figure 9, **L**), epirubicin, and irinotecan, block the replication of DNA by binding to topoisomerase enzymes. Anticancer drugs not included in the above groupings include antineoplastic antibodies and Fc fusion proteins, protein kinase, PI3K and BCL2 inhibitors, asparaginase, HDAC inhibitors, PARP inhibitors, and proteasome inhibitors.

#### Telodendrimers in Drug Combination Therapy

The delivery of bortezomib and doxorubicin has been investigated using a telodendrimer composed of PEG, a segment with diol-containing moieties (caffeic acid, chlorogenic acid, and gluconic acid) used to bind bortezomib via a reversible boronate ester linkage, and a lysine-based dendritic block with Rhein moieties (cassic acid, an anthraquinone), to enhance the affinity for doxorubicin by π-π stacking. It was found that telodendrimers with conjugated bortezomib, upon self-assembly, could load doxorubicin in micelles of sizes 20–30 nm. After accumulation in the tumors by the EPR effect, bortezomib and doxorubicin could be released under acidic conditions. The formulation was tested on mice with SKOV-3 xenograft tumors, and it enhanced the anticancer effect compared to the free bortezomib and Doxil, and their combinations [192].

A series of telodendrimers based on PEG and cholesteryl-functionalized PAMAM dendrons have also been prepared. By varying the cholesteryl functionalities from 1, 2, 4, and 8, it was found that the derivative with eight cholesteryl units had the best loading capacity for DOX and PTX. The micelles were tested for co-delivery of DOX and PTX, and compared to micelles loaded with the individual drug alone. The micelles with both drugs had a higher killing efficiency against HepG2 and MCF-7 cells, compared to micelles loaded with only DOX or PTX at the same concentration, suggesting a synergistic effect of anticancer drugs in the co-loaded system [193]. 

The cisplatin/PTX combination has also been investigated in a telodendrimer based on PEG containing a lysine segment functionalized with succinic acid to bind cisplatin, and a poly(lysine)-based dendron, functionalized with cholic acid to form the hydrophobic block. It was self-assembled into micelles, and loaded with PTX and cisplatin. Testing the system on mice with SKOV-3 ovarian cancer xenografts, it was observed that the co-delivery of PTX and cisplatin resulted in efficient tumor targeting, stronger antitumor effect, and decreased cytotoxicity compared to the free drug combination [194].

In general, the dual loading of anticancer drugs looks promising, and taking advantage of the relative ease to incorporate segments or subunits in the telodendrimer architecture makes these systems capable of carrying a broad range of compounds. This is further highlighted by examples in imaging and pharmacokinetics studies. A telodendrimer composed of a PEG block and a poly(lysine) dendron decorated with cholic acid was used to prepare micelles loaded with both iron oxide nanoparticles for magnetic resonance and Nile red for fluorescent imaging, in a dual-modality nanoparticle system. The size of the micelles was 33.8 ± 5.8 nm, and the MRI sensitivity was found to be high. The in vitro experiments on Raw 264.7 cells showed rapid uptake [195]. In another study, a PEG-poly(lysine)-cholic acid telodendrimer was marked with ^125^I, and their micelles were loaded with ^14^C-labelled paclitaxel. It was reported that this formulation was potentially superior to Taxol in terms of solubility, pharmacokinetics, and tumor accumulation, and it may be useful for both tumor imaging and chemotherapy [196].

Disulfide cross-linked micelles, based on PEG-poly(lysine) and cholic acid with incorporated cysteine moieties for reversible intra-micellar disulfide cross-linking, were loaded with either PTX or AZD9291, and the combination of these two telodendrimers in a mixed glutathione-responsive formulation exhibited a strong synergistic effect in vitro and in vivo [197]. This suggests that two or more drugs do not necessarily have to be loaded into the same telodendrimer-based nanoformulation to take advantage of the combination therapy.

### 4.7. Computer Modeling in Telodendrimer Design and Optimization

Considering the large number of possible variables in the overall composition of telodendrimers, computer-assisted modeling may be used to aid and accelerate the design process. In a study of a series of PEG-poly(lysine) telodendrimers, in which the cholic acid moieties were functionalized with varying numbers of glycerol groups, computer simulations were used to optimize the conformation of subunits of telodendrimers, as well as to optimize substitutions to investigate membrane interactions. The telodendrimers and their membrane activities were found to correlate well with the calculations [198]. In another study, a combination of computer modeling with experimental investigations was used to design an optimal delivery system for PTX, and to analyze the role of the individual subunits in a telodendrimer, based on PEG-poly(lysine), decorated with cholic acid moieties. Multiple simulation studies were conducted, and the results were found to be in agreement with the experimental data, in terms of parameters, such as the average size, drug-telodendrimer stoichiometry, and drug loading capacity [199]. Based on these results and to design a telodendrimer capable of delivering DOX, it was found that changes in subunits of the architectural polymer can have a profound effect on the size, morphology, and asphericity of the micelles [200].

### 4.8. Unique Structures and Applications of Telodendrimers

**Figure 10:**
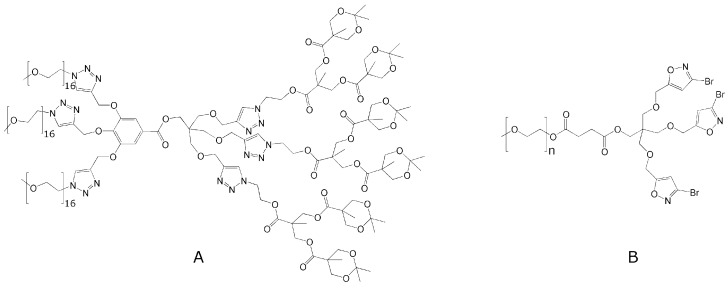
Structural outline of two architectural polymers. (**A**) A poly(ester) dendron and three PEG units [10]; and (**B**) PEG-isoxazole-based telodendrimer [202].

As noted in the examples above, one of the most widely studied architectural polymers is based on PEG-dendron; however, there exists a range of other telodendrimers with more creative designs and applications. In one study, the architectural design challenged the basic build-up with more than one PEG polymer chain attached to the dendritic block, and a series of telodendrimers with one, two, or three PEGs as the hydrophilic block, and a poly(ester) bis-MPA)-based dendron was prepared (Figure 10A). It was reported that that these telodendrimers self-assemble into soft nanoparticles of varied stabilities, which were dependent on the number of PEG arms, with the system containing three PEG units being the most stable. The loading and encapsulation efficiencies were also dependent on the number of PEG units [10]. In most studies, it is the structure of the dendritic block that is altered to examine the relationship between the telodendrimer structure and properties. The results from this study demonstrated that the structure of the hydrophilic polymer block is also important when designing telodendrimers for drug delivery.

A unique functionalization in which dendrons composed of poly(ester) made from 2,2-bis(hydroxy methyl) propionic acid, functionalized with a short PEG containing a guanidine moiety at the terminal end, was used to attach to PEG at its both ends. These were mixed with clay nano sheets, sodium poly(acrylate), and water to form hydrogels with self-healing properties. The PEG functionalized at only one end did not form hydrogels, indicating that cross-linking of the clay nano sheets was important [201]. 

Another interesting telodendrimer, reported recently, is based on a PEG hydrophilic chain and a small isoxazole-based dendron as the hydrophobic block (Figure 10B). This architecture was not used as a carrier for drugs, but since isoxazole is an important part of a wide range of pharmaceuticals, it was interesting to combine this with hydrophilic PEG and test this hybrid for biological activity. It was found that U251N glioblastoma cells were eliminated when exposed to this construct. This type of structure build-up could expand the scope of isoxazole-based systems in combination therapies [202]. 

A telodendrimer composed of PEG as the linear block and a polyester dendritic block prepared from poly(2,2-bis(hydroxy methyl) propionic acid), and functionalized with all-trans-retinoic acid, was used to investigate the delivery of PTX. Upon self-assembly, it formed micelles of ~28 nm diameter with a PDI of 0.103, and a CMC of 3.48 mg/L, with a drug loading capacity of 20% *w/w* [203]. In another example, a telodendrimer containing the well-known PEG-poly(lysine) structure build-up, but decorated with cholic acid and vitamin E, was used to optimize the delivery of gambogic acid for colon cancer treatment. It was found that this formulation is a promising alternative to the more traditional treatments of colon cancer [204].

A telodendrimer was used to support a membrane protein (mMOMP), in order to increase solubility, and to avoid mis-folding of the protein. The MOMP is a membrane protein expressed by *Chlamydia*, which has been shown to be an effective vaccine antigen. Its use is limited by poor stability. PEG-cholic acid-based telodendrimer–nanolipoprotein particles were used in this study, and the cell-free system expressed mMOMP multimers, similar to the native MOMP protein, demonstrating that this approach using telodendrimers can be used to support antigens, which is otherwise difficult [205].

The preparation of nanolipoprotein particles can be cumbersome in producing monodisperse populations. Telodendrimers were investigated in assisting their preparation, and it was found that PEG-poly(lysine)-cholic acid telodendrimers could be used in the cell-free synthesis of lipoproteins, and it resulted in hybrid particles with lower polydispersity [206]. In another interesting study, telodendrimers based on PEG-poly(lysine) containing cholic acid and/or cholesterol were investigated for the delivery of proteins across the blood–brain barrier. Using bovine serum albumin functionalized with fluorescent dye Cy5, it was found that telodendrimers assisted and improved the delivery of the protein into the brain [207]. The results are interesting, and suggest that the telodendrimer can overcome significant challenges in drug delivery across the blood–brain barrier.

A series of telodendrimers were prepared to investigate the effect of surface charge on the in vivo biodistribution of nanoparticles. Positively charged telodendrimers were synthesized through functionalization with lysine, and the negative charges were introduced through aspartic acid. It was found that liver uptake was high for charged telodendrimer derivatives, and slightly negatively charged telodendrimers showed low liver and high tumor uptake [208].

A few examples of telodendrimers with porphyrin in the skeleton have been reported. A PEG-poly(lysine) telodendrimer functionalized with cholic acid and pyropheophorbide (a porphyrin analog) was synthesized, and the resulting micelles with sizes of about 21 nm could chelate a range of metal ions, including Cu(II), Pd(II), Gd(III), and Ga(III) [209]. A similar telodendrimer has been reported for synergistic combination therapy against prostate cancer [210]. 

## 5. Conclusions and Future Perspective

**Figure 11:**
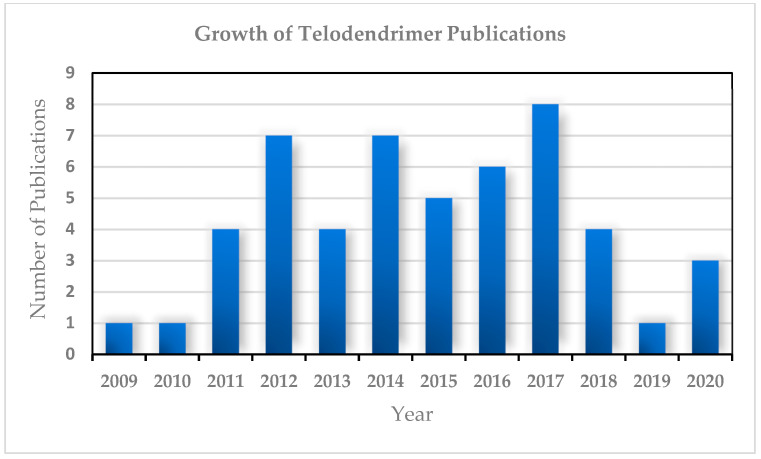
Brief analysis of publications on telodendrimers from 2009–2020 [211]. Note: Publications in 2020 are not complete at this stage.

Delivering active pharmaceutical agents to disease sites is a complex problem, and using nanocarriers to address issues related to drug absorption, distribution, bioavailability, and reducing the metabolic transformation of agents, continues to be a topical area of research. Through significant advances in synthetic methodologies, the structural complexity has evolved from linear di-block amphiphilic polymers to branched (miktoarm stars) and hyperbranched (dendrimers) macromolecules. Much is understood about the aqueous self-assembly of linear amphiphilic di-block copolymers into soft nanoparticles, and physical encapsulation of lipophilic drugs into the core of these micellar nanoformulations. Spherical and monodisperse dendrimers with high surface areas have facilitated drug conjugation through a variety of high-efficiency functionalization methodologies. The ultimate goal of combining the advantageous properties of these two types of macromolecules into a single scaffold has given great impetus to the design of hybrid polymeric structures. Often referred to as telodendrimers, these architectural polymers are relatively new, and understandably their potential has not been fully exploited. One of the most extensively investigated compositions of telodendrimers is that of polyethylene glycol-lysine dendron, in which a significant effort has been made to vary the telodendrimer subunits and functionalization. It is through the investigation of these and other telodendrimers that some limited understanding of the effect of the structure of the dendron on the micelle size, stability, drug loading capacity, and targeted release has been obtained. There is much to be done to elaborate this highly promising platform, and it will come through developing simple and highly versatile methodologies to create variations in both hydrophilic and hydrophobic components. Since the appearance of the first reports on these hyperbranched dendron-linear polymer hybrids several years ago, much of the emphasis has been on exploiting their potential in drug delivery. Based on a brief survey of the literature from 2009–2020 (Figure 11), it is clear that there has been a slow growth in the number of publications in this area [211].

Considering that only a limited number of research groups have been involved in their investigations, telodendrimers may still be considered at the early stages of development. PEG is the most studied linear component of the architectural polymer, but several others, including polysaccharides, could be incorporated into these structures. Telodendrimers containing more than one PEG unit present an interesting variation, which needs to be explored further in expanding the scope of architectural polymers. If structure-property relationships using a large variety of linear and dendron components can be understood, there is a lot to benefit in applying these nanostructures in drug delivery. Considering that we have a plethora of promising drug candidates, it is also becoming evident that in fine-tuning the telodendrimer architecture, and our efficacy in therapeutic interventions, the structure of active agents should also be taken into consideration. Combination therapy presents a very promising protocol in dealing with high morbidity rate diseases, and the inherent composition of telodendrimers can make a great contribution in enhancing our efficacy in drug resistance. Conjugating the drug to the surface of dendrons is easy, but building an understanding of its facile release using biological cues, will enhance the efficacy and scope in drug combination therapy. Telodendrimer are macromolecules of great potential in biological applications, and much progress is yet to be made through tuning and expanding our fundamental knowledge base of the effect of their build-up on the physicochemical properties, and those of their nanoformulations.
